# Synthesis of novel antibacterial nanocomposite CuO/Ag-modified zeolite for removal of MB dye

**DOI:** 10.1038/s41598-023-40790-6

**Published:** 2023-09-11

**Authors:** Nabil A. A. Yahya, O. M. Samir, S. Al-Ariki, Amira A. M. Ahmed, Mohamed A. Swillam

**Affiliations:** 1 Department of Physics, School of Sciences and Engineering, American University in Cairo, New Cairo, 11835 Cairo Egypt; 2 Physics Department, Thamar University, 87246 Thamar, Yemen; 3 Physics Department, Faculty of Science, Ibb University, Ibb, Yemen; 4 Aljanad University, Taiz, Yemen

**Keywords:** Chemistry, Chemical physics, Nanoparticles, Antimicrobial resistance, Photocatalysis, Porous materials

## Abstract

Novel CuO/Ag nanocomposites added zeolite (CAZ) were successfully fabricated, and their effectiveness as an antibacterial on *S. aureus* and MB removal was evaluated. EDX, XRD, and FTIR confirm the presence of the elemental compositions of CAZ. Friable CuO nanorods (10–70 nm in diameter) existed on the surface of the zeolite. Pure zeolite had a higher band gap (5.433 eV) and lower MB removal efficiency than CAZ. The adsorption method by CAZ was more effective at removing MB than photodegradation. 0.10 CAZ had the highest removal effectiveness (~ 99%) and adsorption capacity (~ 70.4 mg g^−1^) of MB. The inhibitory zone diameter for 0.005 CAZ against *S. aureus* was 20 mm, while 0.01 CAZ had a diameter of 17 mm. Azithromycin, ceftriaxone, and erythromycin antibiotics demonstrated lower or no efficacy against *S. aureus* than CAZ. Significant antibacterial activities and wastewater treatment were achieved by CAZ. The combination of photodegradation and adsorption enhanced pollutant removal. It will be interesting to study further the optimal molar ratio for MB removal (0.10 CAZ) in future investigations.

## Introduction

Large quantities of stable industrial effluents with high organic dye content continue to be a significant barrier to achieving clean water using traditional methods. Most dyes are toxic and commonly used in coloring industrial products like leather, cosmetics, textiles, drugs, paper, and printing inks. The majority of dye binds to the surfaces of products during the dyeing process, and the rest is released into surface water (rivers and lakes) without treatment, resulting in polluted water^1,2^. The discharge of dyes into surface water has an impact on the photosynthesis process of aquatic plants, which is one of the most significant food sources for living things there. Moreover, dyes negatively impact human and animal health because they are toxic, carcinogenic, and resistant to oxidizing and biodegrading agents^3^. Consequently, polluted water must be treated before being discharged into the waste stream. However, dyes appear to be challenging to remove from wastewater using traditional methods due to their relative stability and high-water solubility. Several methods have been used to remove pollutants from wastewater, with adsorption and photocatalytic degradation being the most commonly used. The adsorption method is inexpensive, simple, and effective for a wide range of dye concentrations^4,5^. Methylene blue (MB), methyl orange (MO), malachite green (MG), congo red (CGR), and rhodamine B (RHB) are examples of organic dye pollution^5,6^.

Nanomaterials have been widely researched for their capability to remove dyes from wastewater and eliminate bacteria. CuO and Ag have been synthesized as nanoparticles or nanocomposites and examined to use as photocatalysts, antimicrobials, anticancer**,** solar cells, and gas sensors, among other applications^7–11^. CuO is a p-type semiconductor with a small band gap (*E*_*g*_ = 1.2 up to 1.9 eV), low-cost, and non-toxic nature. It has been used to enhance the photocatalytic activity of some wide-band gap semiconductors^12^. Silver and copper are among the most important toxic elements for gram positive and negative bacteria^13,14^. Furthermore, Ag nanoparticles in Na^+^-Y-zeolite and Cu^2+^ nanoparticles in X-zeolite have excellent antibacterial activity^15,16^. The polypyrrole-Ag/graphene/O-carboxymethyl chitosan nanocomposite and polypyrrole-Ag NPs have shown excellent antibacterial action, successfully inhibiting the growth of *E.coli* and *S. aureus* bacteria^17,18^. Electrostatic interactions allowed the cationic Ag nanoparticles to bind to the bacterial surface^19^. Ag nanoparticles can interact with the cytoplasm of *E. coli* and *S. aureus* bacteria and destroy them^20^. Different silver nitrate molar ratios have been examined in Ag/zeolite nanocomposite, and Ag (0.004 M)/zeolite showed the best photocatalytic activity for dye removal and good antibacterial activity^21^. CuO has also been synthesized with several composite materials, including zeolite/Fe_3_O_4_, TiO_2_, WO_3_/CdS, and Ce/Zn, and demonstrated excellent photocatalytic activity for producing hydrogen and dye pollution removal^12,22–26^. Generally, nanoscale materials have a higher specific surface area than that in their bulk. The higher surface area makes available more atoms with a reduction in coordination and can cause rapid agglomeration. Because of agglomeration, the exceptional properties of nanoparticles such as catalysts may be lost. Therefore, zeolites are used as host matrices and supported for nanoparticle applications such as dye photodegradation and adsorption in addition to being a catalyst^27–30^. Zeolites interact with other materials on their surfaces and throughout the bulk. Among all the available natural materials adsorbents, zeolite is commonly used for the removal of toxic pollutants from water because of its low-cost, negative charge, open structure, and high ability to exchange ions^3,31^. Zeolites are microporous aluminosilicate crystalline materials formed of silicon, aluminum, and oxygen in a framework. According to the ratio of aluminum and silicon, there are several kinds of zeolites such as A-zeolite, β-zeolite, X-zeolite, and Y-zeolite. Zeolites have exceptional chemical and physical properties due to their unique structure and act as adsorbents, antibacterial agents, and molecular sieves (for separation)^32–34^. The materials that destroy bacteria without being toxic to surrounding tissues or the environment are known as antibacterial agents. They have a variety of uses, including the filtration of bacterially polluted water, the packaging of food and medicine, and the production of antibacterial textiles.

Some adsorbents have low adsorption capabilities and can only be used under certain circumstances for the removal of dyes^30^. Also, the existence of antibiotic resistant bacteria is one of the biggest challenges to universal health because of their growing resistance to various antimicrobial substances^13^. Besides that, several components can be added with particular advantages to improve the dye removal and antibacterial performance of a single component. Therefore, it would be interesting to investigate the effects of CuO/Ag nanocomposite, CA added zeolite, Z (CAZ) on dye removal in addition to inhibiting or destroying the bacteria.

In this work, CAZ were successfully fabricated by a simple technique, co-precipitation as adsorbents, photocatalytic, and antibacterial. The photocatalytic activities of CAZ were estimated through the degradation of toxic dye (methylene blue, MB) under various UV irradiation times. MB causes eye burns, shock, dyspnea, increased heart rate, cyanosis, vomiting, nausea, diarrhea, and tissue necrosis^3,35^. Moreover, the potential of CAZ as an adsorbent for MB removal in the dark from polluted water was studied. The antibacterial activities of CAZ were estimated by using bacteria of *Staphylococcus aureus* (*S. aureus*), which usually exist in wastewater^16^. The antibacterial effects of azithromycin, ceftriaxone, and erythromycin antibiotics were compared with CAZ. We investigated the impacts of several molar ratios of Cu^2+^, C (0.005, 0.01, 0.05, and 0.10 M), AgNO_3_, A (0.004 M), and zeolite, Z (0.5 gm) contents in CAZ. Pure Z was also examined for comparison. We characterized CAZ by SEM, TEM, EDX, and XRD techniques to investigate their morphology, elemental compositions, and structure. The optical properties of the prepared samples were characterized. The optical gap energies of samples were estimated. Spectra of FTIR and TGA of CAZ were also recorded.

## Experimental procedures

### Materials

Zeolite mordenite, sodium (SiO_2_:Al_2_O_3_ mole ratio 13:1, Alfa Aesar), Methylene blue (MB), (C_16_H_18_ClN_3_S, Sigma-Aldrich), Silver nitrate (AgNO_3_, Sigma-Aldrich, 99.98%), Copper (II) nitrate. (Cu(NO_3_)_2_.2.5H_2_O, 98+%, Chem-Lab NV), Sodium hydroxide (NaOH, Sigma-Aldrich, ≥ 98% ).

### Synthesis of CAZ

**Scheme 1:**
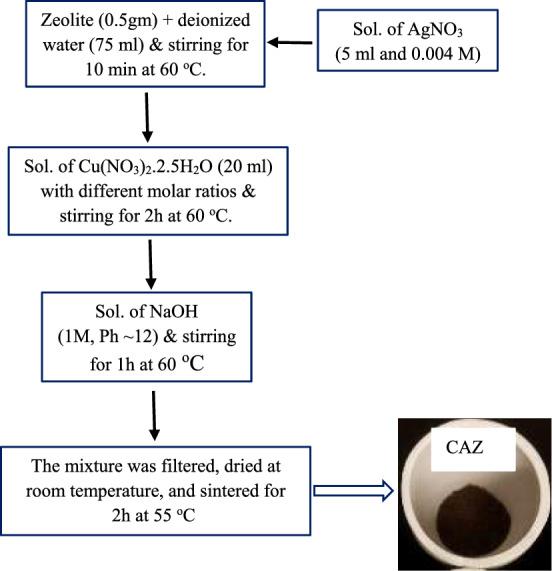
Schematic illustration for CuO/Ag nanocomposite added zeolite mordenite preparation.

CuO/Ag nanocomposite added zeolite mordenite (CAZ) was synthesized by the co-precipitation technique^29,36^. Constant concentrations of AgNO_3_ (0.004 M) and zeolite (0.5 gm) with a different molar ratio of Cu (NO_3_)_2_.2.5H_2_O (0.005, 0.01, 0.05, and 0.10 M) were combined to obtain 0.005, 0.01, 0.05, and 0.10 CAZ. Zeolite powder was suspended in deionized water (75 ml) and stirred for 10 min at 60 °C. AgNO_3_ solution (5 ml and 0.004 M) was slowly added to the zeolite solution and stirred for a further 10 min. Following that, Cu(NO_3_)_2_.2.5H_2_O (20 ml) with different molar ratios was slowly added. The mixture was stirred for 2 h (500 rpm) at 60 °C, followed by the slow addition of NaOH (1M, Ph ~ 12), and left to stir for a further 1 h at that temperature. The mixture of CAZ was filtered and dried at room temperature. The obtained samples (0.005 CAZ, 0.01 CAZ, 0.05 CAZ, and 0.10 CAZ) were heated in a furnace for 2 h at 550 °C. A schematic illustration of CuO/Ag nanocomposite added zeolite is shown in Scheme 1.

### Adsorption and photocatalytic activity experiment of CAZ

The photocatalytic and adsorption activity of CAZ were examined by reducing methylene blue (MB, 10 ppm) in deionized water at room temperature. 0.14 gm of CAZ with different molar ratios of Cu^2+^ (0.005 CAZ, 0.01CAZ, 0.05 CAZ, and 0.10 CAZ) were added to the polluted water (50 ml). The suspension was stirred at room temperature in the dark for 30 min and then under UV irradiation (*λ* = 365 nm) for 30, 60, 90, 120, and 150 min for adsorption and photodegradation tests. Every 30 min, ~ 7 ml liquor was collected and centrifuged (7000 rpm, 20 min) to remove the solid CAZ catalyst particles. By using a UV/VIS spectrophotometer at *λ* = 664 nm, the filtrates were analyzed. The MB removal efficiency (*Reff.*) and adsorption capacity (*Ac*) of samples were obtained by the following eqations^5^.

1 $${{Reff}}{. }\;{{(\% ) = }}\frac{{{\text{C}}_{{0}} - {\text{ C}}_{{\text{e}}} }}{{{\text{C}}_{{0}} }}{{\times 100}}$$

2 $${\textit{Ac}} \left( {{\text{mg g}}^{{ - {1}}} } \right) = \frac{{{\text{C}}_{{0 }} - {\text{ C}}_{{\text{e}}} }}{m} V$$

where ${\text{C}}_{0}$, ${\text{C}}_{\text{e}}$, and *V* are the initial and final concentrations and volume of MB solution, respectively; *m* is the weight of CAZ.

### Antimicrobial activity experiment of CAZ

Stock cultures of *Staphylococcus aureus* were defined by specialists at the medical lab department, Aljanad University for Science and Technology, Taiz, Yemen. These were kept on nutrient agar at 4 °C. A loopful of cells from the stock cultures was transferred to a tube containing 1 ml of ordinary saline for bacteria, which was cultured for 24 h at 37 °C to create the active cultures for the tests. The McFarland 0.5 turbidity level was then attained by diluting the cultures with ordinary saline^37^.

Mueller–Hinton agar aliquots of 20 ml were put into sterile Petri plates. Using a sterile cotton swab, the isolates and standardized bacterial stock suspension were streaked on Mueller Hinton agar medium plates after being adjusted to 0.5 McFarland. A disc of 3 mm diameter was put on sterile Mueller–Hinton agar plates. Each disc was filled with 500 µg of the chemical agents tested. The plates were incubated at 37 °C for 24 h. The diameters of inhibitory zones were measured using a ruler in millimeters^38^.

### Characterizations

The crystalline structure of pure Z and CAZ was analyzed by X-ray powder diffraction (XRD) using Bruker, D8 Discover with CuKα radiation (λ = 1.5406 Å). The field-emission scanning electron microscope (FESEM, Leo Supra 55), transmission electron microscope (HR-TEM, Talos F 200i) and energy-dispersive X-ray (EDX, JCM-6000 Plus) spectroscopy were used to observe the morphology and estimate elemental compositions of the samples, respectively. Spectra of UV–visible absorbance and Fourier transform infrared (FTIR) of samples were recorded using spectrophotometer Lambda 950 and Nicolet 380, respectively. Thermal gravimetric analysis (TGA) of samples was recorded using LTG-A10, LABTRON. PL spectra of samples were investigated using PL system S/N: 1001.

## Results and discussion

### Structural of CAZ

**Figure 1:**
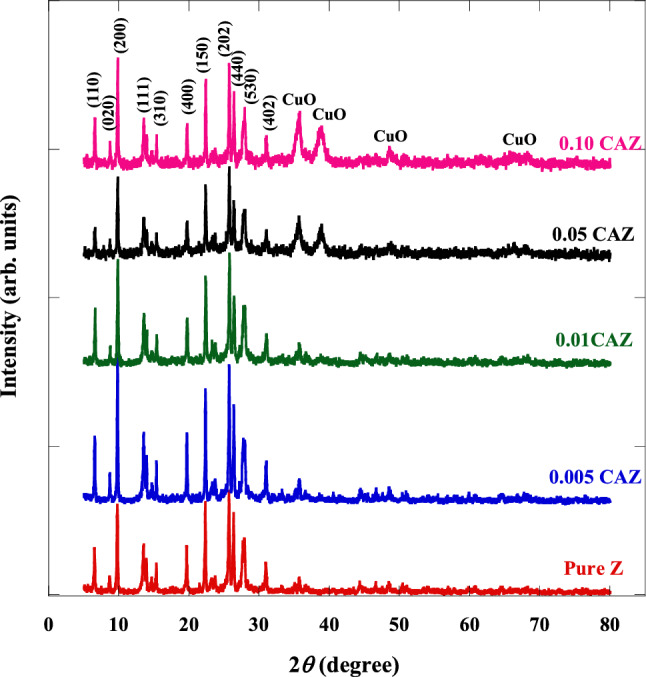
XRD patterns of the pure Z and CAZ with various Cu^2+^ molar ratios.

The X-ray diffraction patterns for the pure Z and CAZ with different Cu^2+^ molar ratios are shown in Fig. 1. The pattern peaks of the pure Z correspond to the zeolite mordenite, sodium orthorhombic structure (PDF#80-0642). XRD patterns confirmed that the added samples kept the zeolite mordenite crystalline structure as a major phase. CAZ has diffraction peaks at *2θ* = 6.50°, 8.61°, 9.75°, 13.41°, 13.82°, 14.56°, 15.27°, 19.59°, 22.19°, 25.55°, 26.23°, 27.83°, and 30.82°. These peak positions correspond to the planes of (110), (020), (200), (111), (130), (021), (310), (400), (150), (202), (440), (530), and (402), respectively. Also, a small number of peaks corresponding to nano CuO (PDF# 02–1040) were observed in the added samples (CAZ) at *2θ* = 35.8°, 38.7°, 48.5°, 66.2°, and 68.4° as a secondary phase. The peak of a low Ag molar ratio (0.004 M) did not appear. MDI Jada 6 software and the following Scherrer’s equation^39^ were used to determine the crystalline size (C*s*) of CuO.

3 $${\text{C}}s =\frac{{0.94} \,\lambda }{{B}_{hkl }{\,\cos}\theta }$$

where λ (1.5406 Å) of XRD), ${B}_{hkl}$ is FWHM, full width at half maximum intensity and $\theta$ is the diffraction peak angle (Bragg’s angle) in radians. The average crystallite size (Cs) of CuO in 0.05 CAZ and 0.10 CAZ was 55 nm.

### Morphology and elemental compositions of CAZ

**Figure 2:**
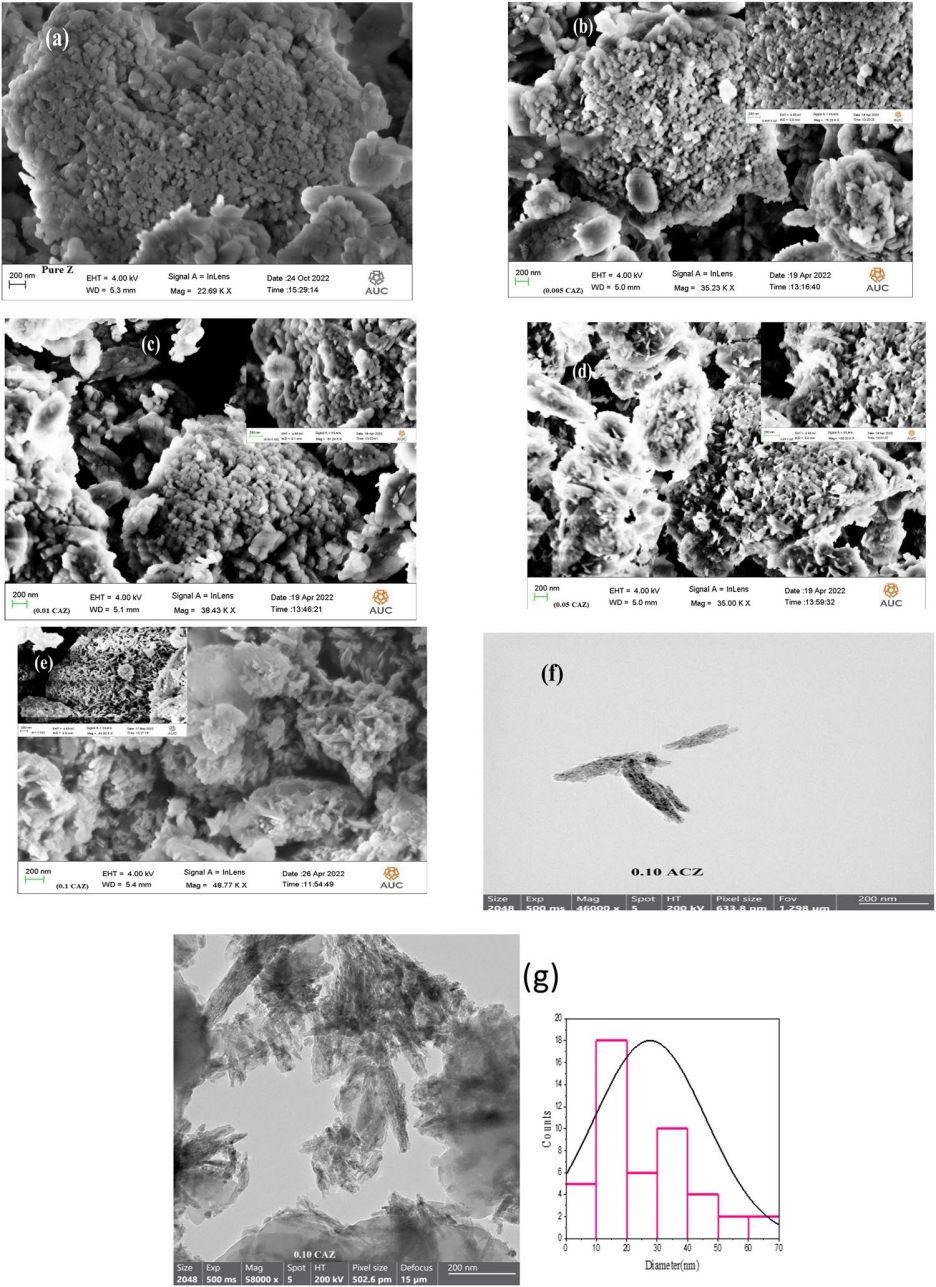
FESEM micrographs of (**a**) pure Z, (**b**) 0.005 CAZ, (**c**) 0.01 CAZ, (**d**) 0.05 CAZ, and (**e**) 0.10 CAZ and TEM images and average diameter of CuO nanorod in 0.10 CAZ (**f**,**g**).

**Figure 3:**
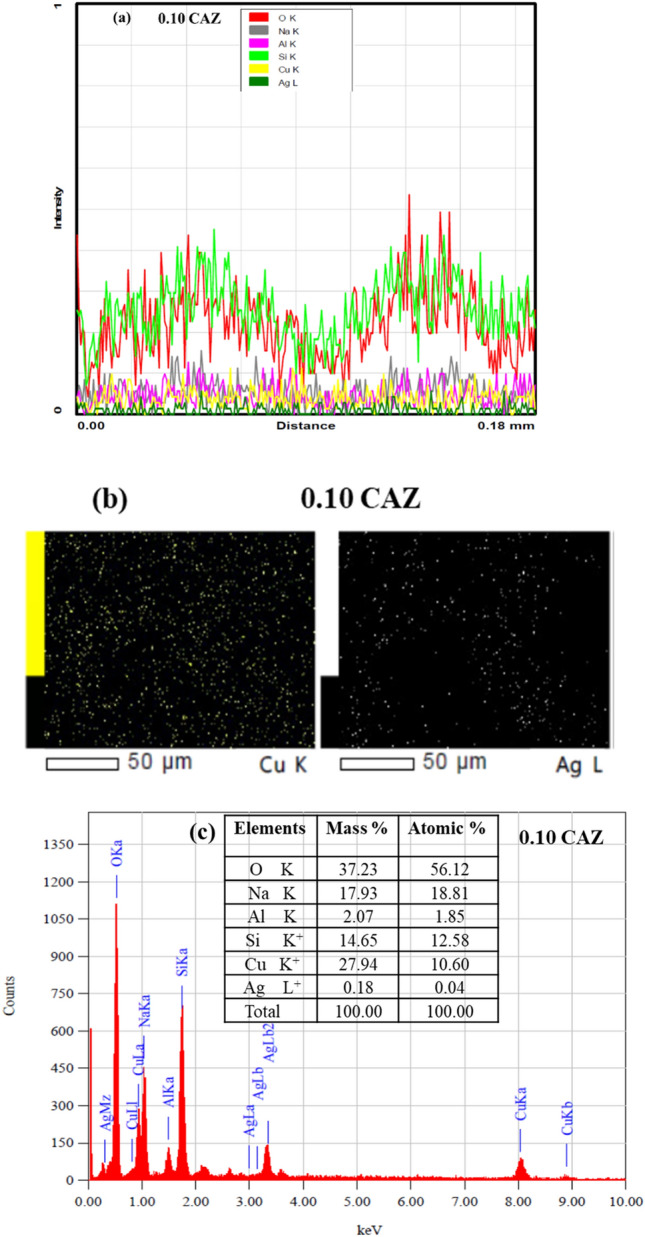
EDX results (**a**) analysis profile of elemental compositions of 0.10 CAZ (O, Na, Al, Si, Cu, and Ag), (**b**) elemental mapping of Cu and Ag nanostructure in 0.10 CAZ and (**c**) spectrum and Table of the atomic percentage of elements.

FESEM images of CuO/Ag nanocomposite added Zeolite with different Cu^2+^ molar ratios are shown in Fig. 2a–e. All samples have spherical, continuously interwoven grains, which are typical of the zeolite phase. Friable CuO nanorods existed in huge numbers on the surface of zeolite with large Cu^2+^ amounts (0.05 and 0.10 M). Nano CuO was also described as having a nanorod-like structure with those of other studies^10,40^. The increase of CuO nanorods peak in the X-ray diffraction patterns confirmed the results shown in Fig. 2d,e. TEM provided additional confirmation existence of CuO nanorods. TEM image shows that CuO nanorods in 0.10 CAZ (Fig. 2f,g) have an average diameter of ~ 30 nm. Spherical Ag nanoparticles were believed to form on CuO nanorods^10^ as shown in Fig. 2f. EDX analysis was carried out and its results confirmed the chemical compositions of all samples. Figure 3a shows the line EDX analysis profile of O K, Na K, Al K, Si K, Cu K, and Ag L, which are the elemental compositions of 0.10 CAZ. Figure 3b shows the elemental EDX mapping of Cu and Ag nanostructures in a 0.10 CAZ sample. Moreover, the EDX spectrum and table for the weight and atomic of 0.10 CAZ confirmed the presence of Cu nanorods and Ag nanoparticles as shown in Fig. 3c. The peaks corresponding to the binding energies of Cu nanorods are shown at around 1, 8, and 9 keV, while the peak at around 0.5 keV corresponds to the binding energy of O^41^. However, the peaks at about 0.25 keV and between 3 to 3.5 keV signified the binding energies of Ag nanoparticles^42^.

### FTIR and TGA analysis

**Figure 4:**
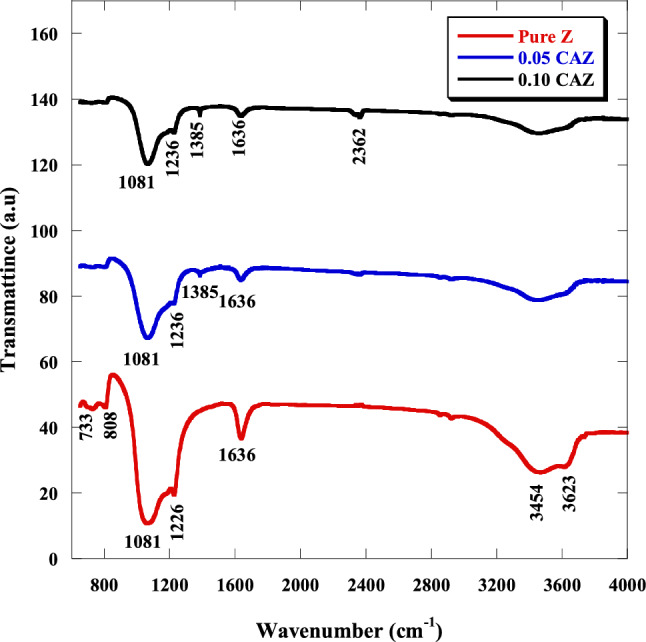
FTIR spectra of the pure Z, 0.05 CAZ, and 0.10 CAZ.

Figure 4 shows FTIR spectra of the pure Z, 0.05 CAZ, and 0.10 CAZ were recorded between 400 and 4000 cm^−1^. The spectra of added samples are almost similar to that of the pure Z. A new peak appeared at ~ 1385 cm^−1^ in the spectra of added samples (0.05 CAZ and 0.10 CAZ) belonging to CuO, respectively^43^. Ag peak with a low molar ratio (0.004 M) did not appear in the FTIR spectra. The vibrations bands in the 3454–3623 are attributed to the hydroxyl group, OH^44, 45^, whereas the peaks between 1600 and 3700 cm^−1^ are attributed to the zeolitic water. The peak of O–Al–O was observed at ~ 1081, while the O–Si–O peak was presented at ~ 700 cm^−1^. The located peak at 1636 cm^−1^ belongs to Si–OH vibration.

**Figure 5:**
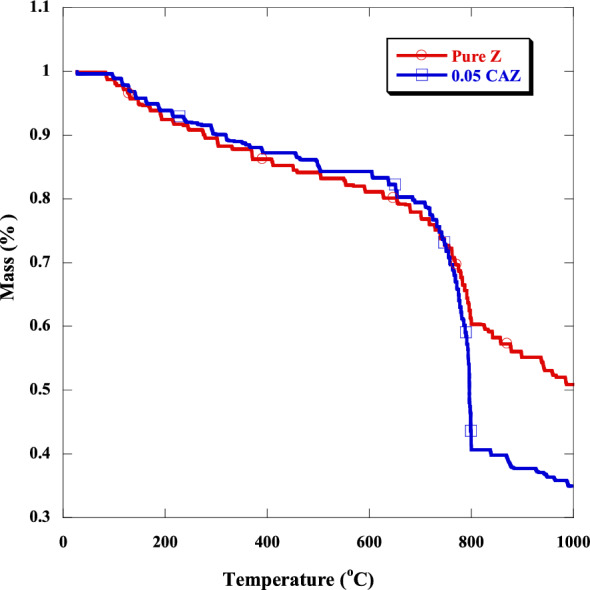
TGA spectra of the pure Z and 0.05 CAZ.

TGA analysis was used to investigate the thermal stability of added samples and compare them with pure zeolite. Figure 5 shows the curves of mass loss versus the temperature of pure Z and 0.05 CAZ. The prepared samples explained three stages of mass loss in the range between 80 and 1000 cm^−1^. The initial degradation process was in the range between 80 and 700 °C because of the loss of physical water and chemically absorbed water evaporation. The major thermal degradation process (second stage) was in the range of 700– 800°C. This can be explained by separating the macromolecular framework and forming stable metal oxalate intermediates^46^. The third stage was in the range higher than 800 °C due to more decomposition of metal oxalates and the formation of metal oxide. The thermal degradation of pure and added zeolite started at around 80 °C and continued up to 1000 °C. The mass loss was about 48.4% for the pure Z and 64.7% for 0.05 CAZ. It was clear that the pure sample had higher thermal stability than the added samples.

### Optical properties of CAZ

The optical characteristics of zeolites have been investigated in a few earlier research. Natural zeolites recorded a band gap energy lower than the standard aluminosilicate zeolites (7 eV)^47^. For example, the direct and indirect energy gaps for natural zeolite clinoptilolite were 4.46 and 4.26 eV, respectively^48^. The band gap of CuO nanoparticles with zeolite was 4.35 eV^43^. A theoretical energy gap value for an idealized clinoptilolite (44 Si and 76 O atoms) was 5.593 eV^49^.

**Figure 6:**
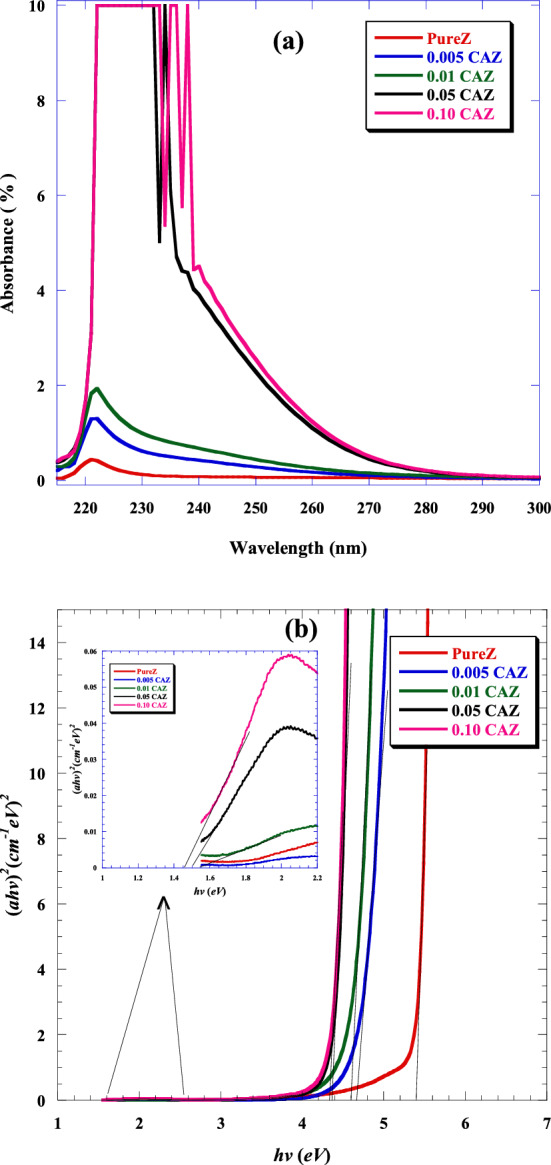
(**a**) UV–visible absorbance and (**b**) plot of ${\left(\alpha hv\right)}^{2}$ versus photon energy (*hν*) of pure Z and CAZ with different Cu^2+^ molar ratios.

In this study, the optical absorptions of pure Z and CAZ were examined at room temperature in the wavelengths between 200 and 850 nm through UV–visible spectrophotometer. As shown in Fig. 6a, the high absorbance of pure Z and CAZ was in the UV region. Pure Z and small amounts of Cu^2+^ in Z (0.005 CAZ and 0.01 CAZ) exhibited a high absorption beak in the range of ~ 221.5 nm and had similar absorbance spectra. However, the maximum absorption peak of a large amount of Cu^2+^ (0.05 and 0.10 M) was observed at 233 nm and 237 nm, respectively. The existence of CuO in/on zeolite may clarify the absorption peaks increased with the increasing molar ratio of Cu^2+^ and extended to a wider wavelength (smaller band gap) compared to the pure Z. Large concentrations of Cu^2+^ (0.05 and 0.10 M) displayed an extra absorbance peak at 238 and 240 nm, respectively, which could be attributed to CuO nanorods^8^. The direct transition band gap energy (*E*_*g*_) was determined using Tauc's equation ^39^.

4 $$\left(\alpha h v\right) = { B}{\left({{hv}} \, -{{E}}_{{g}}\right)}^{{n}}$$

where ${{h}}$ is the Planck constant, $v$ is the wave frequency, *n* = 1/2, is the type of transition, $B$ is a constant, and $\alpha$ is the absorption coefficient. The optical energy gap can be obtained by plotting ${\left(\alpha{hv}\right)}^{2}$ vs. photon energy (*hν*) and extrapolating the linear part near the onset on the curve to the axis of *hv*. Figure 6b shows the band gap energies of samples. The band gaps of CAZ (0.005, 0.01, 0.05, and 0.10 M) were 4.66 ± 0.1, 4.61 ± 0.1, 4.38 ± 0.08, and 4.33 ± 0.07 eV, respectively. Pure Z had a higher band gap (5.433 ± 0.15 eV) than CAZ. The band gap of CAZ was reduced as a result of the possibility of band overlap between CuO/Ag and pure Z. The early band gap energy values were almost similar to those of the pure Z and CAZ. The inset of Fig. 6b displays that the band gaps of CuO/Ag nanocomposite in 0.01CAZ, 0.05 CAZ, and 0.10 CAZ were 1.55 ± 0.03, 1.5 ± 0.02, and 1.45 ± 0.02 eV, respectively. The band gap of CuO/Ag decreased as the Cu^2+^ molar ratio increased. That could be explained as the result of structural deformation, in which Ag ion was replaced by Cu^2+^ in the CuO lattice. These values were consistent with the results of other research^8, 40^.

### Adsorption and photocatalytic activity of CAZ

**Figure 7:**
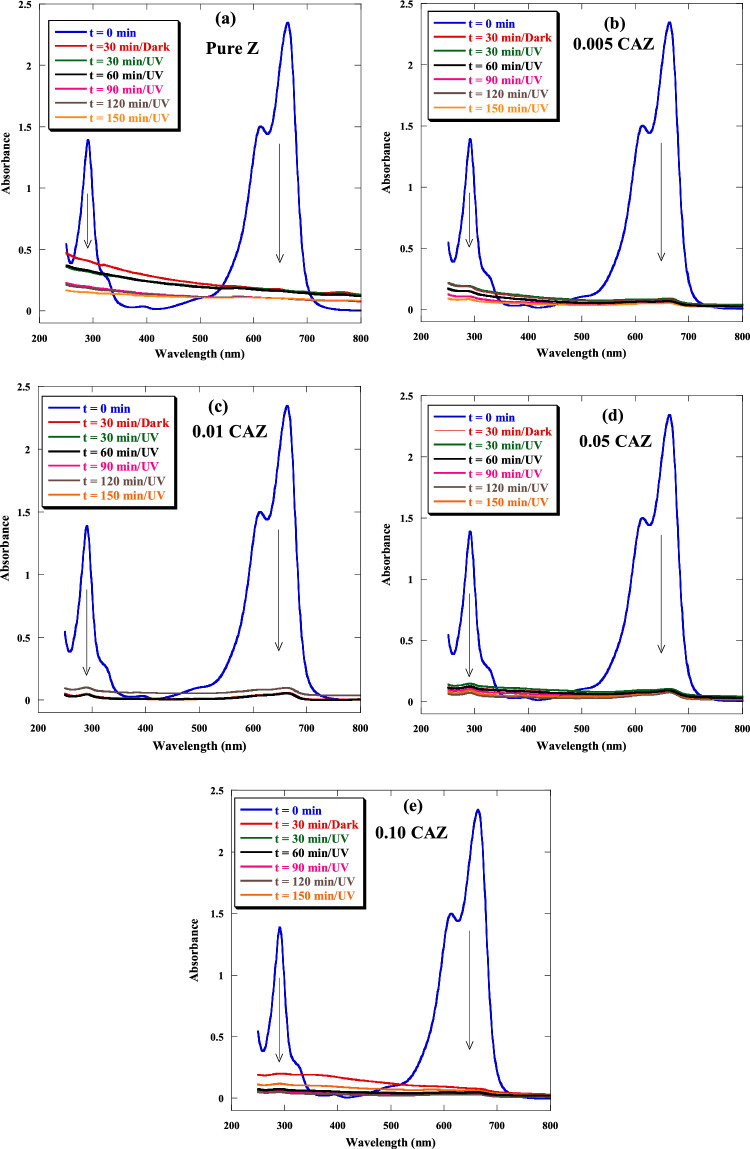
The absorption intensity of MB by (**a**) pure Z, (**b**) 0.005 CAZ, (**c**) 0.01 CAZ, (**d**) 0.05 CAZ, and (**e**) 0.10 CAZ in the dark for 30 min and then under various UV irradiation times.

**Figure 8:**
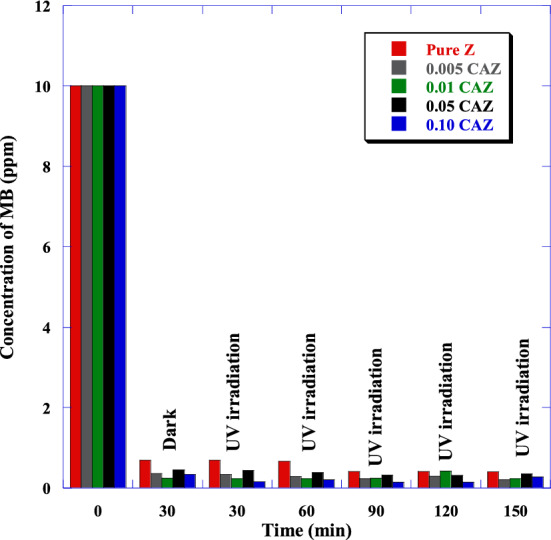
Comparison concentration of MB with the pure Z and CAZ at various Cu^2+^ molar ratios in the dark (30 min) and then under UV light irradiation for 30 up to 150 min.

**Figure 9:**
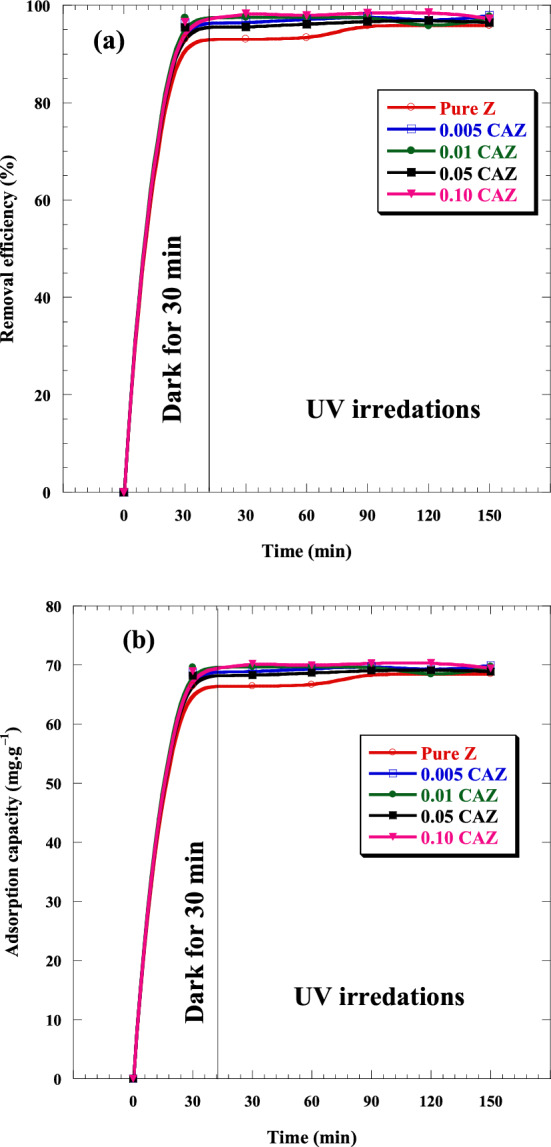
(**a**) Removal efficiency and (**b**) adsorption capacity of MB treated by the pure Z and CAZ with different Cu^2+^ molar ratios in the dark (30 min) and then under UV light irradiation (30 up to 150 min).

The potential of samples as adsorbents for removing MB from wastewater in the dark was investigated. Moreover, photocatalytic activities of the pure Z and CAZ with varying Cu^2+^ molar ratios were also investigated through the degradation of MB under various UV irradiation times. Figure 7a–e show the absorption intensity of MB by the pure Z and CAZ with different Cu molar ratios in the dark and then under various UV irradiation times. The absorbance spectrums of samples showed a significant decrease in the MB intensity within 30 min in the dark and then decreased very slightly when exposed to UV light for 30 to 150 min. Also, CAZ displayed a higher removal MB compared to the pure Z. Figure 8 and Table 1 show the MB concentrations in the initial aqueous solution (0 min) and with the presence of the samples in the dark (30 min) and then in UV light (30 to 150 min). In comparison to pure Z, CAZ had lower MB concentrations in the dark and when exposed to UV light. In the dark, MB concentration decreased significantly from its initial aqueous solution (10 ppm) to 0.70 ppm for pure Z, whereas it was lower for CAZ and reached 0.25 ppm for 0.01 CAZ. However, the lowest MB concentration was 0.15 ppm for 0.10 CAZ under UV irradiation. Figure 9a and Table 2 display the influence of pure Z and CAZ on the removal efficiency of MB (*Reff.*) in the dark and UV irradiation. CAZ was a more effective removal efficiency of MB than the pure Z. A slight increase in the MB removal efficiency was observed when exposed to UV light for longer than 30 min compared with efficiency in the dark. The MB removal effectiveness by pure Z was about 93% in the 30 min dark condition, while CAZ demonstrated removal efficiencies of between 96 and 97.5%. When exposed to UV light for longer than 30 min, pure Z presented removal efficiencies of between ~ 93 and ~ 96%, whereas CAZ exhibited efficiencies of between ~ 96 and ~ 99%. The maximum MB removal efficiency was achieved by 0.10 CAZ with the highest Cu^2+^ concentration and lowest energy gap during 30 min of darkness and 120 min of UV exposure. The adsorption capacity (*Ac*) of pure Z and CAZ to the MB removal is shown in Fig. 9b and Table 3. The adsorption capacity of pure Z was 66.46 mg g^−1^ in the dark and increased to 68.52 mg g^−1^ with increasing the UV irradiation time. However, the adsorption capacity of CAZ was higher than that of pure Z. The adsorption capacity of 0.10 CAZ was up to 70.37 mg g^−1^ (Table 3). MB's maximum adsorption capacity and removal efficiency in the presence of CAZ were more significant than those reported with Fe_3_O_4_@zeolite@CuO^12^, ZnO nanoparticles-HYzeolite^50^, Fe_3_O_4_/zeolite NaA nanocomposite^51^, CuO nanotubes/zeolite^52^, NiFe_2_O_4_/Ag_3_PO_4_^53^, TiO_2_@ZnO nanoparticle^54^, Carbon nanotubes^55^, and Ag-ZnO nanoparticles/S-g-C_3_N_4_^56^ (Table 4).

**Table 1:** Comparison concentration of MB with the pure Z and CAZ at various Cu^2+^ molar ratios in the dark (30 min) and then under UV light (30 up to 150 min).

Sample	MB concentration (ppm)						
	Initial	30 min Dark	30 min UV	60 min UV	90 min UV	120 min UV	150 min UV
Pure Z	10	0.70 ± 0.08	0.70 ± 0.08	0.67 ± 0.08	0.42 ± 0.05	0.41 ± 0.05	0.41 ± 0.05
0.005 CAZ	10	0.37 ± 0.05	0.35 ± 0.05	0.28 ± 0.04	0.23 ± 0.04	0.30 ± 0.01	0.21 ± 0.05
0.01 CAZ	10	0.25 ± 0.08	0.24 ± 0.07	0.24 ± 0.07	0.25 ± 0.07	0.42 ± 0.08	0.23 ± 0.07
0.05 CAZ	10	0.45 ± 0.06	0.44 ± 0.05	0.38 ± 0.05	0.33 ± 0.05	0.31 ± 0.04	0.35 ± 0.04
0.10 CAZ	10	0.35 ± 0.05	0.17 ± 0.02	0.20 ± 0.03	0.15 ± 0.02	0.15 ± 0.02	0.27 ± 0.04

**Table 2:** Comparison removal efficiency of MB by the pure Z and CAZ with various Cu^2+^ molar ratios in the dark (30 min) and then under UV light (30 up to 150 min).

Sample	MB removal efficiency (%)						
	Initial	30 min Dark	30 min UV	60 min UV	90 min UV	120 min UV	150 min UV
Pure Z	0	93.04 ± 0.91	93.04 ± 0.91	93.34 ± 0.93	95.84 ± 0.95	95.91 ± 0.95	95.92 ± 0.95
0.005 CAZ	0	96.31 ± 0.92	96.50 ± 0.96	97.16 ± 0.96	97.66 ± 0.97	96.90 ± 0.96	97.94 ± 0.97
0.01 CAZ	0	97.46 ± 0.96	97.60 ± 0.97	97.64 ± 0.97	97.53 ± 0.97	95.82 ± 0.95	97.66 ± 0.97
0.05 CAZ	0	95.50 ± 0.95	95.60 ± 0.97	96.18 ± 0.97	96.72 ± 0.96	96.87 ± 0.97	96.48 ± 0.96
0.10 CAZ	0	96.54 ± 0.96	98.33 ± 0.98	97.97 ± 0.98	98.46 ± 0.98	98.52 ± 0.98	97.26 ± 097

**Table 3:** Comparison adsorption capacity of MB by the pure Z and CAZ with various Cu^2+^ molar ratios in the dark (30 min) and then under UV light (30 up to 150 min).

Sample	MB adsorption capacity (mg g^−1^)						
	Initial	30 min Dark	30 min UV	60 min UV	90 min UV	120 min UV	150 min UV
Pure Z	0	66.46 ± 0.77	66.46 ± 0.77	66.67 ± 0.77	68.46 ± 0.86	68.51 ± 0.86	68.52 ± 0.86
0.005 CAZ	0	68.79 ± 0.86	68.93 ± 0.86	69.40 ± 0.88	69.76 ± 0.88	69.28 ± 0.97	69.95 ± 0.86
0.01 CAZ	0	69.61 ± 0.77	69.72 ± 0.80	69.74 ± 0.80	69.66 ± 0.80	68.44 ± 0.77	69.75 ± 0.80
0.05 CAZ	0	68.22 ± 0.83	68.29 ± 0.86	68.70 ± 0.86	69.09 ± 0.86	69.19 ± 0.88	68.91 ± 0.88
0.10 CAZ	0	68.96 ± 0.86	70.24 ± 0.88	69.98 ± 0.91	70.33 ± 0.94	70.37 ± 0.94	69.47 ± 0.88

**Table 4:** Comparison of maximum removal efficiency and adsorption capacity for MB dye with some previous studies.

Materials	Removal efficiency (%)	Adsorption capacity (mg/g)	References
Fe_3_O_4_@zeolite@CuO	~ 97	–	^12^
ZnO nanoparticles—HY zeolite	80	–	^50^
Fe_3_O_4_/zeolite NaA nanocomposite	∼96.8	∼40.36	^51^
CuO nanotubes/zeolite	95.9%	–	^52^
NiFe_2_O_4_/Ag_3_PO_4_	92.63	–	^53^
TiO_2_@ZnO nanoparticle	25	–	^54^
Carbon nanotubes	–	64.7	^55^
Ag-ZnO nanoparticles/S-g-C_3_N_4_	97	–	^56^
CuO/Ag nanocomposite/zeolite	~ 99	~ 70.4	This work

### Adsorption and photodegradation mechanism proposed

From the previous radical scavenging experiments^57^, it can be concluded that the main reactive species accountable for breaking down MB are hole (*h*) and superoxide (${\text{O}}_{2}^{*-}$). During the process of photocatalytic degradation of MB using AgBr/Ag_2_CO_3_-zeolite, ${\text{OH}}^{*}$ and *h* were the primary reactive radicals while ${\text{O}}_{2}^{*-}$ had limited influence^58^. The following equations summarize the photodegradation mechanism proposed for degrading MB using CAZ^59^. Electron–hole (*e*–*h*) pairs could be created because the CAZ photocatalysts in the MB solution absorb light energy (*hv*) equal to or higher than their energy gap (*E*_*g*_). Electrons in CuO/Ag could be excited from the valence band to the conduction band, resulting in the immediate production of holes in the valence band (Eq. 5). When an electron and a hole interact with water, they could be produced superoxide (${\text{O}}_{2}^{*-}$) and hydroxyl radicals (${\text{OH}}^{*}$) (Eq. 6 and Eq. 7), respectively. The harmless by-products CO_2_ and H_2_O could be created when the superoxide and hydroxyl radicals interact with the MB dye (Eq. 8).

5 $$\text{Photocatalyst }+ {{hv}}\left({{UV}}\right)\to \, \text{Photocatalyst }[{{e}} \, + \, {{h}}]$$

6 $${{e}} \, +{{O}}_{2}={{O}}_{2}^{*-}$$

7 $${{h}} \, + {O} {{H}}^{-}\to{{OH}}^{* }$$

8 $${{O}}_{2}^{*-},{{OH}}^{*}+ \, {MB \,dye} \, \to \, {{CO}}_{2}+{{H}}_{2}{O }$$

Generally, a large amount of MB pollutant was adsorbed in the dark condition. These can be attributed to the strong electrostatic interactions between the cationic MB and the negatively charged zeolite framework that has a large surface area. Moreover, the attractive morphology and larger surface enhance the MB adsorption by nano CuO/Ag. A high band gap in CAZ, which limits the production of electron–hole pairs, is the cause of their low photodegradation activity for MB. Additionally, the small band gap of nano CuO/Ag causes faster recombination of the electron–hole pairs. Photocatalytic activity can be affected by the band gap and properties of the surface area, structure, and morphology of the catalyst^60^.

### Photoluminescence (PL) examination

**Figure 10:**
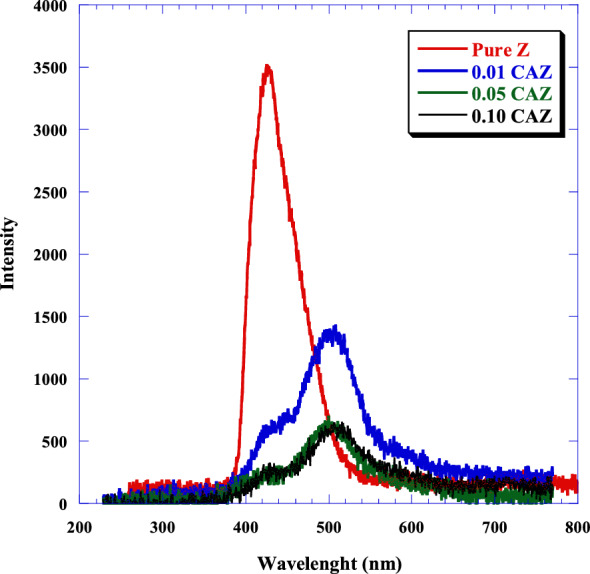
Photoluminescence spectra of pure Z, 0.01 CAZ, 0.05 CAZ and 0.10 CAZ.

The process of photogenerated electron transfer in CAZ can be examined using PL spectroscopy. The rate of recombination for electron/hole (*e*/*h*) pairs was determined during the activation of photocatalysis. Figure 10 shows the PL spectra of pure Z, 0.01 CAZ, 0.05, and 0.10 CAZ excited with a wavelength of 325 nm, within the range 200–800 nm. It's well known that PL spectra with higher photoemission peaks suggest a greater rate of recombination for charge carriers and a lower photocatalytic performance^61^. Pure zeolite displayed a peak at 425 nm and fluorescence intensities greater than 0.01 CAZ, 0.05 CAZ, and 0.10 CAZ. The PL spectra of CAZ samples showed an emission peak at 500 nm. In comparison to pure Z, nanocomposite CuO/Ag-modified zeolite (CAZ) samples were more successful in reducing *e*/*h* pair recombination rates, hence improving photocatalysis. 0.10 CAZ was the most effective at slowing *e*/*h* pair recombination and increasing photocatalysis.

### Kinetic study

A prior study found that the most useful model for determining the catalytic activity of pure MB was the first-order model^53,59^. Consequently, the first-order kinetic model was investigated by the following equation^62^ (Eq. 9) to determine the adsorption and photodegradation kinetic of MB in the presence of CAZ with different Cu^2+^ molar ratios.

9 $$ln\left({\text{C}}_{0}/ {\text{C}}_{\text{e}}\right)={k}_{1}t$$

where ${\text{C}}_{0}$ and ${\text{C}}_{\text{e}}$ are the initial and final concentrations of MB solution, respectively; ${k}_{1}$ is the first-order rate constant, and *t* is the time.

**Figure 11:**
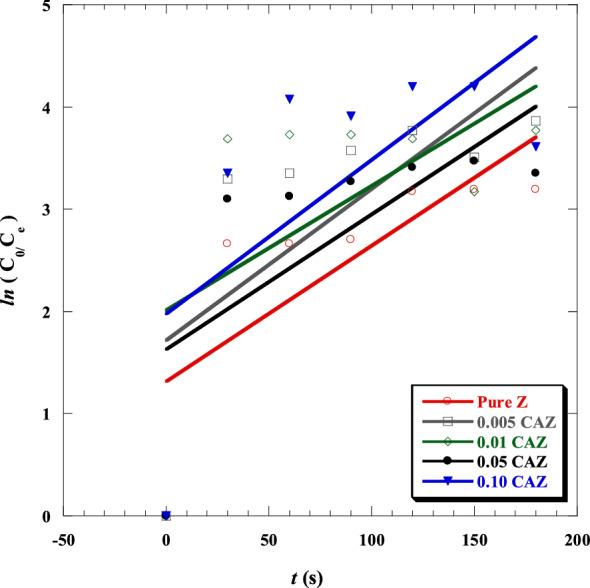
Kinetics of adsorption and photodegradation of MB dye in the presence of the pure Z and CAZ at various Cu^2+^ molar ratios.

Figure 11 shows the curves of $ln\left({\text{C}}_{0}/ {\text{C}}_{\text{e}}\right)$ versus* t* for pure Z, 0.005 CAZ, 0.01 CAZ, 0.05 CAZ, and 0.10 CAZ. The slope of their straight-line fitting was used to determine ${k}_{1}$. The kinetic first-order rate constant ${k}_{1}$ and Pearson's *r* were shown in Table 5. The rate constant of 0.10 CAZ was the highest compared with other samples, indicating that 0.10 CAZ removed MB more effectively. MB dye was more susceptible to oxidative changes in the presence of 0.10 CAZ than the other samples tested.

**Table 5:** The kinetic first-order rate constant ${k}_{1}$ and Pearson's *r* of MB in the presence of the pure Z and CAZ with different Cu^2+^ molar ratios.

Sample	${k}_{1}$	*r*
Pure Z	0.0133 ± 0.005	0.757
0.005 CAZ	0.0148 ± 0.006	0.704
0.01 CAZ	0.0122 ± 0.008	0.569
0.05 CAZ	0.0132 ± 0.006	0.684
0.10 CAZ	0.0151 ± 0.007	0.6491

### Antibacterial activity of CAZ

**Figure 12:**
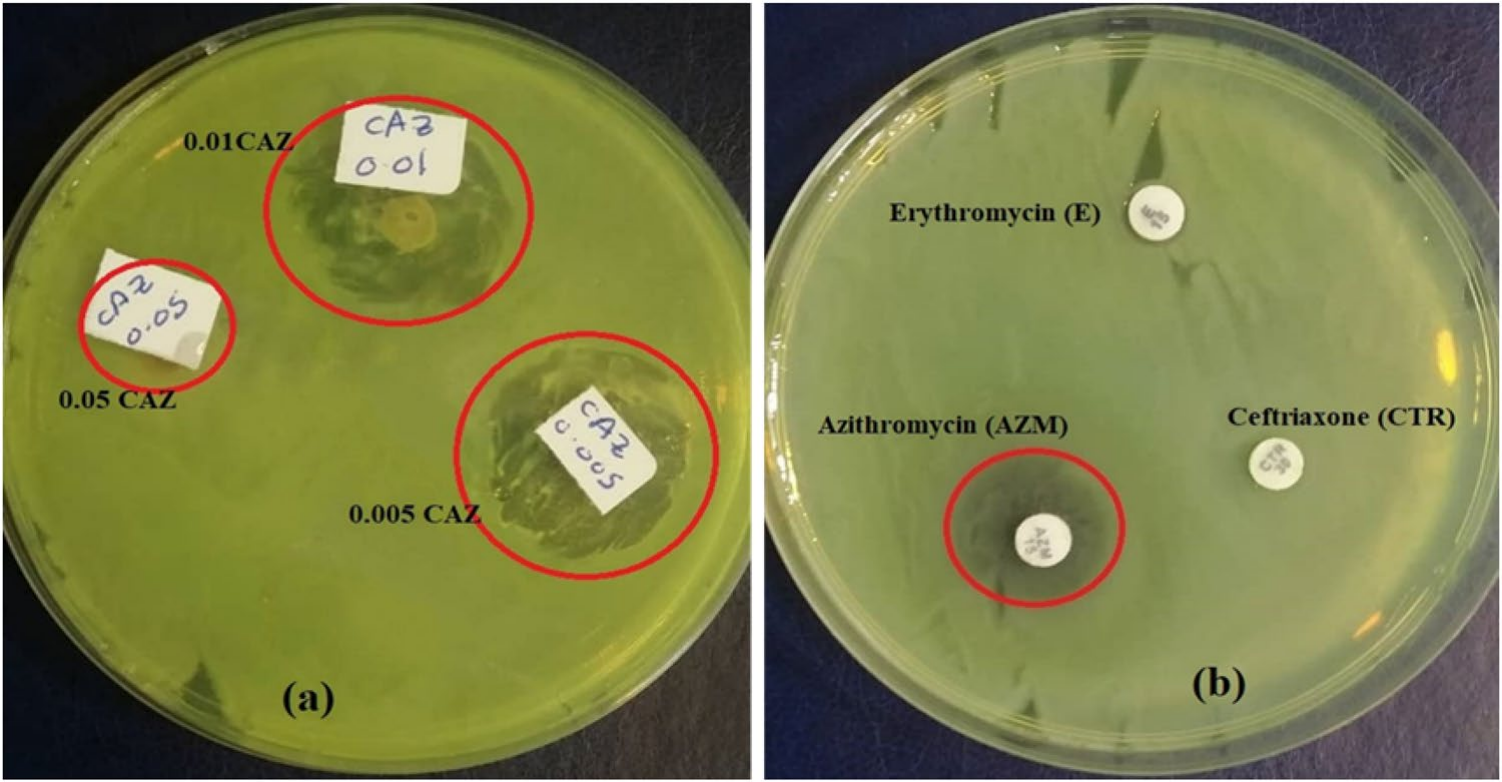
Results of inhibition zone of disc diffusion method for (**a**) 0.005 CAZ, 0.01CAZ, and 0.05 CAZ and (**b**) azithromycin, ceftriaxone, and erythromycin antibiotics against *S. aureus* bacteria.

The antibacterial activity of CAZ on *Staphylococcus aureus* (*S. aureus*, gram-positive) was examined using the disc diffusion technique. The antibacterial performance of CAZ was examined based on the diameter of the inhibitory zone around the tested chemicals. Figure 12a shows the antibacterial effects of 0.005 CAZ, 0.01 CAZ, and 0.05 CAZ against *S. aureus* bacteria. The bacterial inhibition zones indicate that 0.005 CAZ and 0.01CAZ had antibacterial activity against *S. aureus* bacteria. The inhibitory zone diameter for 0.005 CAZ was 20 ± 1.5 mm, while it was 17 ± 0.8 mm for 0.01 CAZ. No bacterial inhibition zones were detected for 0.05 CAZ and 0.10 CAZ. The antibacterial effect of azithromycin (AZM), ceftriaxone (CTR), and erythromycin (E) antibiotics were compared to that of CAZ (Fig. 10b). The inhibitory effect of 0.005 CAZ and 0.01 CAZ against *S. aureus* was more than that of the antibiotics. The azithromycin inhibited *S. aureus* with a 15 ± 0.7 mm inhibition zone, whereas *S. aureus* exhibited resistance to ceftriaxone and erythromycin, which had no inhibition zones. These findings are consistent with other studies, which show that azithromycin works as an antibiotic and an anti-inflammatory against gram-negative, gram-positive, and atypical bacteria^63^. Also, the ceftriaxone and erythromycin antibiotics have less or no antimicrobial activity against the growth of *S. aureus*^64,65^.

## Conclusion

Novel nanocomposite CuO/Ag-modified zeolite (CAZ) with different Cu^2+^ molar ratios was synthesized for antibacterial and dye removal. CAZ was examined for MB removal capability as a model of polluted water and antibacterial activity on *S. aureus* as a model of gram-positive bacteria. The band gap energies of CAZ decreased as Cu^2+^ increased. They were between 4.61 and 4.66 eV, with pure zeolite having a higher band gap (5.433 eV) than CAZ. The removal efficiency and adsorption capacity of MB were higher in CAZ than in pure zeolite. The highest MB removal efficiency of ~ 99% was achieved by 0.10 CAZ. A combination of CAZ photodegradation and adsorption enhanced the efficacy of MB removal. The adsorption process had the most significant effects on the MB removal rate compared to the photodegradation process. *S. aureus* bacteria was inhibited by a 25 mm inhibition zone formed by 0.005 CAZ and a 17 mm inhibition zone formed by 0.01 CAZ. Azithromycin, ceftriaxone, and erythromycin were less effective or ineffective against *S. aureus* than CAZ. These findings demonstrated that CAZ had significant antibacterial properties in addition to its capacity for wastewater treatment. In future work, examining the effect of CuO/Ag nanocomposites addition with different Ag and zeolite concentrations will be interesting.

## Data Availability

The data will be available from the corresponding author upon request.
